# Semaphorin 4C: A Novel Component of B-Cell Polarization in Th2-Driven Immune Responses

**DOI:** 10.3389/fimmu.2016.00558

**Published:** 2016-12-07

**Authors:** Di Xue, Marylin Desjardins, Gabriel N. Kaufman, Marianne Béland, Salem Al-Tamemi, Eisha Ahmed, Shao Tao, Roland H. Friedel, Walid Mourad, Bruce D. Mazer

**Affiliations:** ^1^Translational Research in Respiratory Diseases, The Research Institute of the McGill University Health Center, Montreal, QC, Canada; ^2^McGill University Health Center, Montreal Children’s Hospital, Montreal, QC, Canada; ^3^Sultan Qaboos University Hospital, Muscat, Oman; ^4^Icahn School of Medicine at Mount Sinai, New York, NY, USA; ^5^Department of Medicine, University de Montreal, Montreal, QC, Canada

**Keywords:** Semaphorin 4C, B-cells, immune synapse, Th2 responses

## Abstract

**Background:**

Semaphorins are important molecules in embryonic development and multiple semaphorins have been identified as having key roles in immune regulation. To date, there is little known about Semaphorin 4C (Sema4C) in immune biology. We report for the first time that Sema4C is inducible in human and murine B-cells and may be important for normal B-cell development.

**Methods:**

Human tonsillar B-cells were studied following activation *via* anti-CD40 antibodies in the presence or absence of representative Th1, Th2, and regulatory cytokines. Murine B-cells from WT and Sema4C^−/−^ mice were similarly stimulated. B-cell phenotyping in WT and Sema4C mutant mice was performed by flow cytometry and lymphoid architecture was studied by immunohistochemistry. Sema4C expression and synapse formation were analyzed by confocal microscopy.

**Results:**

Gene array studies performed on human tonsillar B-cells stimulated to produce IgE revealed that Sema4C was among the top genes expressed at 24 h, and the only semaphorin to be increased under Th2 conditions. Validation studies demonstrated that human and murine B-cells expressed Sema4C under similar conditions. Sema4C^−/−^ mice had impaired maturation of B-cell follicles in spleens and associated decreases in follicular and marginal zone B-cells as well as impaired IgG and IgA production. In keeping with a potential role in maturation of B-cells, Sema4C was expressed predominantly on CD27^+^ human B-cells. Within 72 h of B-cell activation, Sema4C was localized to one pole in a synapse-like structure, in association with F-actin, B-cell receptor, and Plexin-B2. Cell polarization was impaired in Sema4C^−/−^ mice.

**Conclusion:**

We have identified a novel immune semaphorin induced in human and murine B-cells under Th2 conditions. Sema4C appears to be a marker for human memory B-cells. It may be important for B-cell polarization and for the formation of normal splenic follicles.

## Introduction

Semaphorins have important roles in neural development and angiogenesis, and have recently emerged as key molecules in immune regulation (1). There are eight classes of semaphorins, of which five are expressed in vertebrate cells. The wide range of expression of these molecules and their binding partners, the plexins and neuropilins, contribute broadly to important homeostatic processes and especially the structural organization of tissues (2). Semaphorins are characterized by a unique seven-branch propeller-like SEMA domain and are expressed as transmembrane molecules, GPI-anchored proteins, or are secreted in soluble forms (3).

B-lymphocytes are crucial players in host defense as producers of antibody and as antigen presenting cells. B-cells also initiate and potentiate allergic pathologies, by the production of IgE and the elaboration of key cytokines that enhance allergic inflammation (4). Our laboratory is particularly interested in the role that B-cells play in autoregulating Th2-mediated inflammation: we were the first to determine that human B-cells produced the key Th2 cytokine IL-13 (5), and identified signaling pathways related to expression of the IL-13R on human B-cells (6). In this context, we undertook to uncover novel molecules induced by activation of B-cells *via* Th2 cytokines, the initiating steps for class switching and production of IgE. Using expression profiling of Th2 stimulated human tonsillar B-cells, we identified multiple genes that were previously known to influence IgE production. Semaphorin 4C (Sema4C) was among the highest expressed genes following B-cell activation and was uniquely expressed at a much higher level than other members of the Semaphorin family.

There are no data to date that implicate Sema4C in immune biology, particularly in B-cells. Semaphorins have been observed to be involved in immune cell trafficking, apoptosis, cell growth, and cytokine production. Specifically, molecules including Sema 3A, 4A and 4D, 6C, 7A, and 6D are involved in T-cell/dendritic-cell interaction, integrin signaling, and T-cell proliferation (7, 8). Sema4D has been studied in the context of B-cell development, autoimmunity, and malignancy (9, 10). Sema4D KO mice have mild deficiencies in B-lymphocytes and antibody production. Sema4A and Sema4D deficient mice have been studied in models of allergic airways disease, but no specific effect has been found on B-cells in these models. In this study, we present data which demonstrate that Sema4C expression is a feature of B-cell activation specifically in Th2 responses.

Sema4C appears to be upregulated in maturing B-cells, and its expression was particularly restricted to human CD27^+^ cells, which denote memory B-cells. To further characterize the known functional protein association networks of Sema4C, we queried the STRING (**S**earch **T**ool for the **R**etrieval of **I**nteracting **G**enes/Proteins) database (version 10.0, available at http://www.string-db.org) (11), which examines known relationships in between genes, building networks of predicted functional associations based on gene ontology (GO) annotations, pathways, and domains (12). The gene network of Sema4C predicted by the STRING database displayed statistically significant enrichments for biological processes including receptor localization to synapse, cell projection organization, semaphorin–plexin signaling pathways, plasma membrane components, and cell junctions. Using Sema4C^−/−^ mice as well as human B-cells, we present evidence suggesting an important role for Sema4C in development of B-cell lymphoid follicles and in antibody production.

## Materials and Methods

### Subject Selection and Ethics Statement

Children between the ages of 3–12 requiring tonsillectomy or adenoidectomy were randomly recruited from the otolaryngology clinic at the Montreal Children’s Hospital as part of a study on B-cell responses to corticosteroids. At tonsillectomy, eligible children were not taking nasal or inhaled corticosteroids. Patient caregivers all provided written informed consent. Patients with immunodeficiency were recruited as part of the Canadian Primary Immunodeficiency Evaluative Survey (C-PRIMES) (13). All human subject protocols were approved by the Research Ethics Board of the McGill University Health Centre.

### Transgenic and Wild-type Mice

Semaphorin 4C heterozygotes (*Sema4C^±^*) were bred on the C57BL/6 background (14). *Sema4C^±^* were interbred with resulting litters consisting of WT, *Sema4C^±^*, and *Sema4C^−/−^*. The *Sema4C^−/−^* mice were identified by genotyping as described previously (14), using a three-primer multiplex PCR with the following primers: TGGTGTGGCTTACCCTGTGCTTTG (genomic forward), AGAAAGGAGCCAGGTTGTTCTGCA (genomic reverse), and ACTTCCGGAGCGGATCTCAAACTC (vector reverse), which amplified a 620 bp wild type and a 430 bp mutant fragment (14). Littermate WT mice were used as control. All animals were housed in a specific pathogen-free environment, and all experiments were conducted in accordance with the regulations and standard guidelines of the Canadian Council on Animal Care, and were approved by the Animal Care Committee of the Research Institute of the McGill University Health Center.

### Human and Murine B-Lymphocyte Preparation and Culture

Human tonsils were minced and resuspended in wash medium consisting of RPMI 1640, 2% FBS (Hyclone, Logan, UT) with 2 mM l-Glutamine, 50 U/mL penicillin, 50 µg/mL streptomycin, 15 mM HEPES, and 0.5 µg/mL amphotericin B (Life Technologies, Mississauga, ON, Canada). The cells were overlaid on density-gradient separation medium (Lymphoprep, StemCell Technologies, Vancouver, BC, Canada) and centrifuged to isolate the mononuclear cells according to the manufacturer’s directions. These cells were mixed with human red blood cells and B-cells were purified using RosetteSep Human B-Cell Enrichment Cocktail (StemCell Technologies, Vancouver, BC, Canada). In some experiments, B-cells were further purified into CD27^+^ and CD27^−^ subsets with the EasySep Human Memory B-Cell Isolation Kit (StemCell Technologies, Vancouver, BC, Canada). Human B-cells were cultured from 1 to 7 days in complete medium consisting of RPMI 1640 with 10% FBS, 2mM l-Glutamine, 50 U/mL penicillin, 50 µg/mL streptomycin, 1mM Sodium Pyruvate, and 15mM Hepes at 5 × 10^5^ cells/mL. The cells were stimulated with or without anti-CD40 (1 μg/mL, purified from the G28.5 cell line) (5), IL-4 (100 U/mL), IL-21 (50 ng/mL) (Peprotech, Rocky Hill, NJ, USA) or IL-13 (100 U/mL), IFN-γ (10 ng/mL), IL-12 (10 ng/mL), or IL-10 (10 ng/mL) (e-Biosource). Cells used for flow cytometry and RNA extraction were cultured in 24-well plates. Cells used for immunofluorescent microscopy were grown in eight-well slide chambers (BD Falcon, Mississauga, ON, Canada).

Peripheral blood mononuclear cells (PBMCs) were isolated from blood of healthy controls or patients with common variable immune deficiency (CVID) using Lymphoprep density gradient (StemCell Technologies, Vancouver, BC, Canada) according to the manufacturer’s instructions. Cells were either cytospun immediately, or grown in eight-well slide chambers in indicated culture conditions for 5 days, at 10^6^ cells/mL, in complete medium with or without anti-CD40 (1 μg/mL), IL-4 (200 U/mL), and IL-21(50 ng/mL). Culture medium was refreshed after 72 h without adding cytokines.

Murine splenic B-cells were purified with the EasySep Mouse B Cell Enrichment Kit (StemCell Technologies, Vancouver, BC, Canada) following the manufacturer’s instructions. B-cells were plated either in six-well plates (Corning Costar, Corning, NY, USA) or eight-well culture slide chambers (Becton Dickenson, Mississauga, ON, Canada) at 5 × 10^5^ cells/mL, stimulated with anti-CD40 antibodies (1 μg/mL, HM40-3, eBioscience, San Diego, CA, USA) in the presence of IL-4 and IL-21 (40 ng/mL, Prospec, East Brunswick, NJ, USA), in complete medium as indicated.

### Gene-Expression Microarray Analyses

RNA was extracted from tonsillar B-cells with TRIzol (Life Technologies). Samples used for microarray hybridization were further cleaned with RNeasy Mini silica-gel membrane columns (Qiagen, Hilden, Germany), and RNA quality and concentration were assessed by Bioanalyzer Microcapillary Electrophoresis (Agilent, Santa Clara, CA, USA). Samples had 260/280 absorbance ratios > 1.8 and RNA Integrity Numbers (RIN) > 7.0. For samples used for RT-qPCR, RNA quality and concentration were assessed by Nanodrop spectrophotometry, and 260/280 absorbance ratios were >1.8.

RNA samples were analyzed using Affymetrix GeneChip Human Genome U133 Plus 2.0 microarrays, which contain probe sets for 54,675 unique gene-expression sequences from the NCBI UniGene database, build 159. This provides genome-wide expression coverage for 24,442 genes. Preparatory cRNA synthesis and labeling, microarray hybridization reactions, and array scanning were performed according to standard protocols at the McGill University and Génome Québec Innovation Centre Microarray core facility. Gene-expression data were subsequently processed using the Bioconductor packages oligo (15) for data read-in and normalization, and limma (16) for linear modeling and differential-expression statistics. Probe intensities were normalized across all arrays by the robust multi-array average (RMA) algorithm (17). Differential gene expression of anti-CD40/IL-4-treated B-cells versus unstimulated B-cells (control group) was calculated by linear modeling of the contrast and empirical Bayes sample variance shrinkage, followed by moderated *t*-tests with false discovery rate (FDR) correction (18). Results were expressed in terms of log(2)-fold change. Differentially expressed genes were defined as genes with log(2)-fold change greater than 2. All array data have been deposited in the GEO database, accession number GSE71810.

### Reverse-Transcription Quantitative PCR

Quantification of the mRNA message coding for Semaphorin 4C (Sema4C) was performed by RT-qPCR. Sema4C primers were synthesized by Invitrogen (Carlsbad, CA, USA). TRIzol-extracted mRNA was prepared as above, followed by reverse transcription with DNase treatment. PCR reactions were performed using the Applied Biosystems 7500 Real-Time PCR system in a volume of 10 µL per reaction, containing 1 μL of cDNA, 0.5 mM of each primer, and 5 μL of QuantiTect SYBR Green PCR mix (Qiagen, Mississauga, ON, Canada). The denaturation and amplification conditions for mRNA were 95°C for 15 min, followed by 55 cycles of PCR. Each cycle included denaturation at 95°C for 20 s, annealing at temperature indicated in Figure S1 in Supplementary Material for 20 s, and extension at 72°C for 30 s. The temperature transition rate was 20°C/s, except when heating at 72°C, when it was 5°C/s.

### Detection of Cell-Surface Phenotype by Flow Cytometry

Mouse spleen and lymph nodes were mechanically dissociated through a 90-mm mesh. Red blood cells were lysed using RBC Lysis Buffer (BioLegend, San Diego, CA, USA) according to the manufacturer’s instructions. Single cell suspensions were resuspended in PBS or complete medium. For flow cytometry, single cell suspensions were labeled with CD19-APC (6D5), CD21-Pacific Blue (7E9), CD23-PE/Cy7 (B3B4), CD93-PerCP (AA4.1), IgD-APC/Cy7 (11-26c.2a), IgM-FITC (RMM-1), CD43-PerCP/Cy5.5 (1B11), and CD138-PE (281-2) (BioLegend, San Diego, CA, USA) for 30 min at 4°C, followed by three washes with PBS. Data were acquired on BD LSR II Flow Cytometer and analyzed using FlowJo software version 10.0 for Macintosh.

### Immunohistochemistry and Microscopy

Confocal microscopy was used to assess expression of Sema4C and its co-localization with molecules on murine and human B-cells. Cells cultured in eight-well slide chambers were fixed with 4% formaldehyde. They were blocked with Protein Block (Dako, Burlington, ON, Canada) for 45 min, and stained with primary antibodies for 1 h, followed by incubation for 1 h with the secondary antibody at room temperature. Nuclei were stained with Hoechst 33342 (Life Technologies, Carlsbad, CA, USA) for 15 min at room temperature, and photomicrographs were taken with a Zeiss LSM780 Laser Scanning Confocal microscope.

Spleens from WT and Sema4C^−/−^ mice were snap-frozen, cryosectioned, and stained for histomorphometric measurement with ImageJ software (https://imagej.nih.gov/ij/).

Antibodies used included anti-mouse Sema4C (R&D Systems), anti-human Sema4C (R&D systems), anti-human Plexin-B2, anti-mouse Plexin-B2 (eBioscience), anti-human IgG (Acris), anti-sheep NL557, anti-sheep NL493 (R&D Systems), anti-mouse Alexa555 (Invitrogen), Phalloidin-Alexa Fluor 488 (Cytoskeleton), Cholera Toxin B-Alexa Fluor 488 (Sigma), and anti-actin (Millipore).

### Lipid Raft Protein Purification

Lipid rafts were isolated according to previously published protocols (19–21). Stimulated and unstimulated isolated murine B-cells (10^8^) were washed twice in cold serum-free RPMI 1640 and lysed in 600 μL of ice-cold TNE buffer (10 mM Tris, pH 7.5, 150 mM NaCl, and 5 mM EDTA) containing 1% Triton X-100, 2 mM Na_3_VO_4_, and protease inhibitors (Roche Molecular Biochemicals, Montreal, QC, Canada) on ice for 30 min. Cell lysates were mixed with an equal volume of 80% OptiPrep (Cosmo Bio, Carlsbad, CA, USA) in TNE and deposited in Beckman ultracentrifuge tubes. The samples were overlaid with 1.8 mL 35%, 1.8 mL 30%, 1.8 mL 25%, and 0.6 mL 20% OptiPrep in TNE and centrifuged for 4 h at 45,000 rpm at 4°C. Eleven fractions (580 μL) were collected and aliquots of each fraction were mixed with 6× Laemmli buffer containing 6% 2-mercaptoethanol and heated for 5 min at 95°C. Samples were resolved by SDS-PAGE, and proteins were transferred onto polyvinylidene difluoride (PVDF) membranes (Bio-Rad, Mississauga, ON, Canada). After blocking with 5% BSA and 0.05% Tween 20 in TBS, the membranes were incubated with anti-mouse Sema4C antibodies (R&D Systems, Minneapolis, MN, USA), washed extensively, and subjected to chemiluminescent detection with HRP-conjugated anti-sheep IgG Ab using ECL (Bio-Rad, Mississauga, ON, Canada).

### Detection of Immunoglobulins by ELISA

Total murine and human IgG and murine IgA from cell-culture supernatants or serum were measured by ELISA according to the manufacturer’s directions (Bethyl Laboratories, Inc., Montgomery, TX, USA). Serum and supernatant IgE were measured according to a previously published method (22).

### Statistical Analysis

All data are presented as means ± SEM. Student’s *t*-test was performed for the quantitative assays. Statistical analysis was performed with Prism 6.0 software (GraphPad, La Jolla, CA, USA). Mann–Whitney tests were performed to compare relative expression of Sema4C and IgG production between CVID subjects and controls. *p*-Values ≤ 0.05 were considered statistically significant.

## Results

### Expression Profile of Human B-Cells Identifies Sema4C as a Th2-Induced Gene

**Figure 1:**
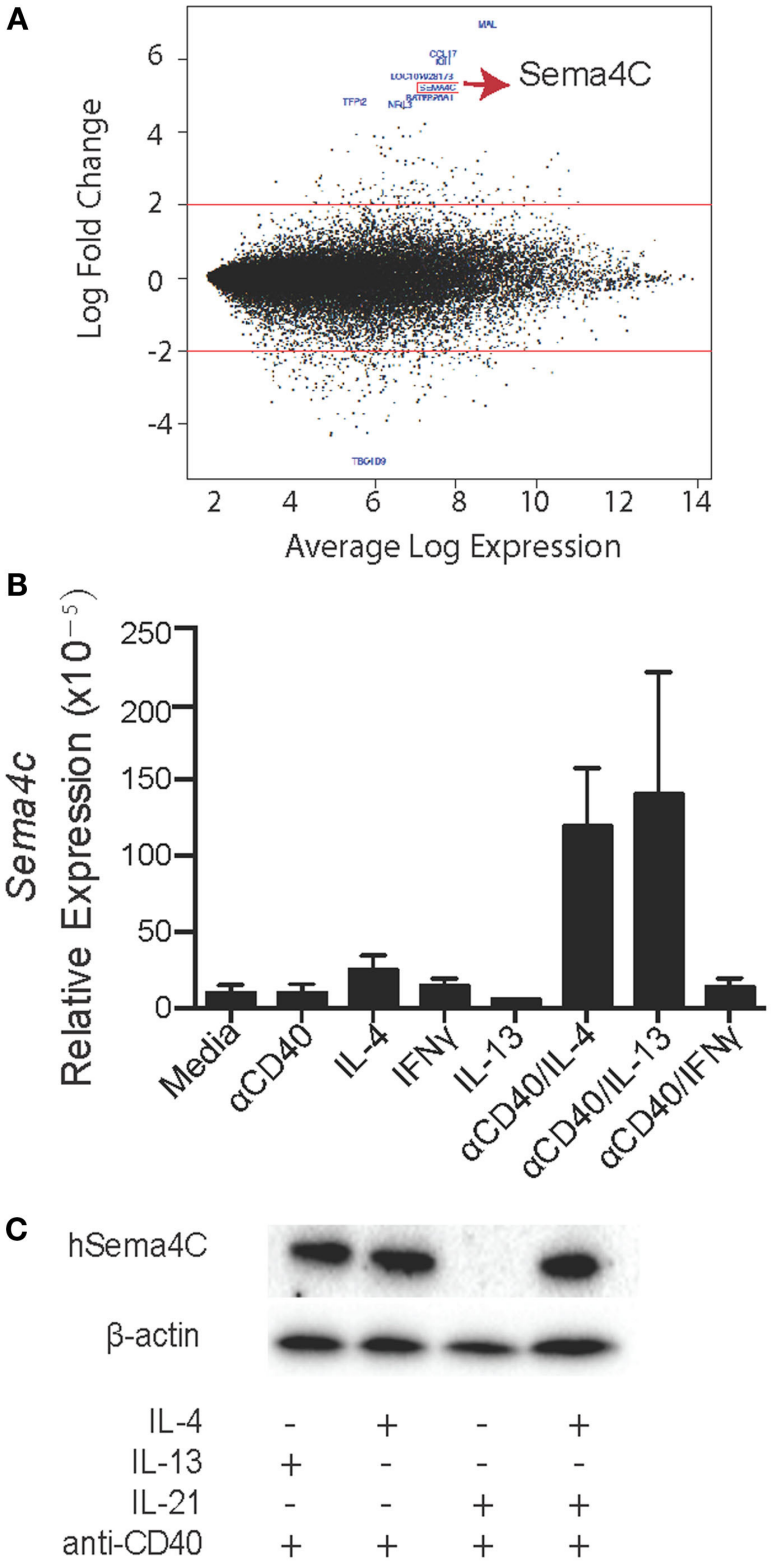
**Expression profile of human B-cells identifies Sema4C as a Th2-induced gene**. **(A)** As part of a larger study on human allergic B-cell responses, human tonsillar B-cells were isolated by RosetteSep Human B-Cell Enrichment Cocktail, followed by anti-CD40 + IL-4 stimulation. After 24 h, RNA was collected from stimulated cells. RNA samples were analyzed using Affymetrix GeneChip Human Genome U133 Plus 2.0 microarrays. M–A [minus log(2)-fold-change versus average log-expression] plot for the contrast of anti-CD40 + IL-4 versus unstimulated B-cells. Red lines indicate the threshold of twofold change for differential gene expression. The 10 most differentially expressed genes are labeled with their NCBI gene symbol (GEO accession number: GSE71810). **(B)** Real-time PCR was performed to validate the microarray results. Human Sema4C mRNA was detected in tonsillar B-cells stimulated with indicated stimulators for 24 h. mRNA samples were normalized to RPL27 as a reference gene. **(C)** Western blot was performed to confirm human Sema4C protein level in B-cells stimulated with indicated stimulators for 5 days. Means ± SEM, *n* = 10, *p < 0.05.

Highly purified B-lymphocytes were isolated from five atopic and five non-atopic randomly chosen children undergoing tonsillectomy as part of a study on B-cell responses to corticosteroids following exposure to Th2 cytokines. Purified B-cells were stimulated with anti-CD40 and IL-4 for 24 h, and mRNA was analyzed by gene chip analysis. Among the highest expressed genes at 24 h of stimulation, as detected by the Affymetrix U133 Plus 2.0 array, was Sema4C (Figure 1A; Table 1). Sema4C was upregulated by 5.2-fold (log base 2) (Table 2) compared to unstimulated B-lymphocytes. This was detected equally in atopic and non-atopic children. Table 2 indicates changes from baseline for other known members of Semaphorin families. There were insignificant changes relative to control for Sema4A, B, D, and F. Indeed, as seen in Table 2, other Sema molecules, both soluble and transmembrane, did not appear to be readily induced by Th2-mediated stimulation in human B-cells.

**Table 1:** **Top 10 differentially expressed genes in anti-CD40/IL-4-treated B-cells versus unstimulated cells**.

Gene symbol	Log(2)FC	Average expression	*t*	*p*-Value	FDR *p*-value	*B*
MAL	6.938443644	8.781046141	22.12378951	3.63E − 11	2.84E − 07	15.3252569
CCL17	6.105803764	7.680392611	16.5397378	1.11E − 09	2.28E − 06	12.50870126
IGH	5.935820046	7.673584573	12.06096389	4.14E − 08	1.94E − 05	9.187431123
LOC101928173	5.515918425	7.149173386	11.68877045	5.88E − 08	2.39E − 05	8.850838852
SEMA4C	5.21704198	7.547023138	21.29194618	5.71E − 11	3.47E − 07	14.97365834
TBC1D9	−5.036190035	5.841642242	−15.44879776	2.45E − 09	3.26E − 06	11.80732796
CYP26A1	4.930913543	7.459197163	7.998347059	3.54E − 06	0.000373054	4.810661778
BATF3	4.918258573	7.085415104	22.83952642	2.49E − 11	2.27E − 07	15.61211466
TFPI2	4.822924251	5.491276439	16.39571399	1.23E − 09	2.31E − 06	12.41949125
NFIL3	4.719930309	6.618679958	37.8227258	5.99E − 14	3.27E − 09	19.4033636

*Log(2)FC: log(2)-fold change for contrast; average expression: average log2-expression for the probe over all arrays and channels; *t*: moderated *t*-statistic; *p*-value: raw *p*-value; FDR *p*-value: adjusted *p*-value using false discovery rate multiple comparisons correction; *B*: log-odds that gene is differentially expressed*.

**Table 2:** **Differential expression of Semaphorin family members**.

Gene symbol	Log(2)FC	Average expression	*t*	*p*-Value	FDR *p*-value	*B*
SEMA4C	5.21704198	7.547023138	21.29194618	5.71E−11.	3.47E−07	14.97365834
SEMA4B	−1.235599024	6.345794409	−5.3118569	0.000178806	0.005258783	0.830235823
SEMA4D	−0.896259857	6.432564626	−4.467420803	0.000751329	0.013817334	−0.629351612
SEMA7A	0.403151491	6.68879853	2.173788882	0.05024346	0.23154331	−4.774256708
SEMA4A	0.38441167	6.470459825	1.141283825	0.275807098	0.603021278	−6.246174802
SEMA6A	0.29074576	4.455055136	2.122703445	0.055054311	0.244902321	−4.859130022
SEMA4F	−0.262174692	4.964741139	−1.400057281	0.186578356	0.493907796	−5.935875619
SEMA6B	−0.227714401	5.232972907	−1.912644868	0.079719927	0.306604318	−5.197925052
SEMA5B	0.181312147	3.816976678	1.187470534	0.257800641	0.583543996	−6.194386687
SEMA3A	−0.160932883	2.718524137	−1.478466849	0.164803331	0.462079013	−5.832748049
SEMA3C	0.149658539	2.86694811	1.384716973	0.191108118	0.500118045	−5.955582839
SEMA6D	0.148505863	3.514224396	1.797559595	0.097195351	0.345193007	−5.375636572
SEMA3D	0.139936085	2.544893601	1.543396954	0.14843985	0.436662717	−5.744425555
SEMA6C	0.125123198	4.331553392	1.234448712	0.240442023	0.563560149	−6.140054909
SEMA3B	0.119732353	3.229057118	0.848524468	0.412596128	0.724463832	−6.534159586
SEMA3F	0.111764922	4.607073365	0.693642806	0.500988306	0.787113093	−6.656077523
SEMA5A	−0.092673159	3.234224403	−0.901608551	0.384837035	0.703311324	−6.487356543
SEMA3E	−0.049619278	2.151782378	−0.650234658	0.527677768	0.803799681	−6.686208382
SEMA4G	0.042891245	4.635360403	0.236850745	0.816729696	0.93973818	−6.878921767
SEMA3G	0.006477205	3.8604494	0.035343716	0.972381279	0.99116659	−6.908249422

*Probe with highest differential expression is shown*.

*Log(2)FC: log(2)-fold change for contrast; average expression: average log2-expression for the probe over all arrays and channels; *t*: moderated *t*-statistic; *p*-value: raw *p*-value; FDR *p*-value: adjusted *p*-value using false discovery rate multiple comparisons correction; *B*: log-odds that gene is differentially expressed*.

### Th2 Specificity of Sema4C Expression on Human B-Cells

We validated the expression of Sema4C on B-lymphocytes, including determination if the induction of Sema4C was a general function of B-cell activation or was Th2 specific. Using RT-qPCR, we found that there was minimal detectable Sema4C mRNA following stimulation with either anti-CD40 antibodies or IL-4 alone. Indeed, baseline detection of Sema4C mRNA in human B-lymphocytes was low, with significant increases in expression following anti-CD40 + IL-4 stimulation (Figure 1B). The combination of anti-CD40 and IL-13 induced expression of Sema4C to a similar degree as IL-4. Culture with anti-CD40 antibodies in the presence of Th1 cytokines IFN-γ or IL-12, or the T-regulatory cytokine IL-10 did not induce Sema4C mRNA (Figure S1 in Supplementary Material). Similarly, expression of Sema4C protein was restricted to the combination of anti-CD40 and IL-4 or IL-13 (Figure 1C) but not with anti-CD40 in combination with IFN-γ, IL-10, or IL-12 (Figure S1 in Supplementary Material). Increases in protein expression were seen with the combination of IL-4 and IL-21 as well, but IL-21 alone did not induce Sema4C protein in the absence of IL-4 (Figure 1C).

### Sema4C^−/−^ Mice Have Decreased B-Cells in Secondary Lymphoid Organs and Abnormal Splenic Organization

**Figure 2:**
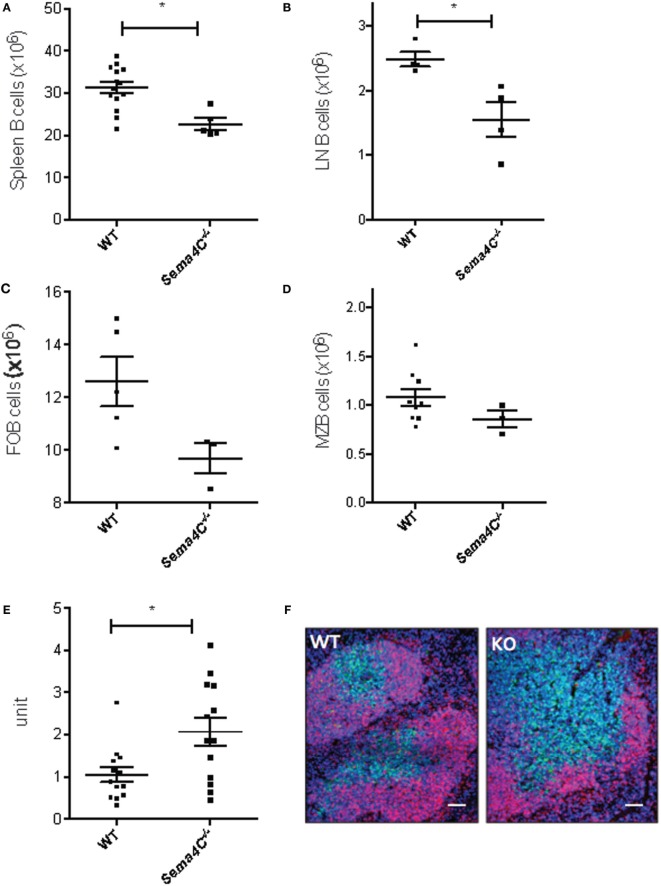
**Sema4C^−/−^ mice have decreased B-cells in secondary lymphoid organs and abnormal splenic organization**. **(A–D)** Sema4C^−/−^ mice had decreased B-cells in secondary lymphoid organs. Total B cell counts in spleen **(A)** and cervical lymph nodes **(B)**, follicular B-cells **(C)** and marginal zone B-cells **(D)** from naïve Sema4C^−/−^ or littermate WT mice were determined by flow cytometry. B-cells were defined as CD19^+^CD3^−^, follicular B-cells: CD19^+^CD93^−^CD21^−^CD23^+^IgM^Low^IgD^High^, marginal zone B-cells: CD19^+^CD93^−^CD21^+^CD23^−^IgM^High^IgD^Low^. **(E,F)** Sema4C^−/−^ mice exhibit abnormal secondary lymphoid organ follicular structure. **(E)** Size of splenic follicles in naïve Sema4C^−/−^ or WT mice, measured by ImageJ. Each dot indicates one follicle and follicles were randomly selected from five different mice. *n* = 5. **(F)** Representative immunofluorescence images of spleens from naïve Sema4C^−/−^ or WT mice stained for CD19 (red), CD3 (green), and nuclei (blue). Data shown are representative of five mice. Images showing Sema4C^−/−^ mice had larger follicles as well as poor demarcation between T- and B-cell margins. Scale bar: 200 μm.

To delineate if Sema4C played a role in B-cell development, we obtained Sema4C^−/−^ mice bred on the C57BL/6 background (14). Viable Sema4C^−/−^ mice did not have any overt phenotypic abnormalities, and did not display spontaneous increased susceptibility to infection. There were no significant differences in the number of pro-, pre-, and immature B-cells detected in the bone marrow of Sema4C^−/−^ compared to WT mice. Peripheral blood lymphocyte subsets were also similar in Sema4C^−/−^ and WT (data not shown). However, Sema4C^−/−^ mice had 30% fewer B-cells in spleens and lymph nodes compared to WT (Figures 2A,B), but exhibited no differences in number of CD4 T cells, CD4^+^ regulatory T cells, CD8 T cells, dendritic cells, macrophages, monocytes, eosinophils, or neutrophils (Table 3). We further evaluated B-cell subpopulations in the spleen, including marginal (CD19^+^CD21^+^IgM^High^IgD^Low^CD23^−^CD93^−^) and follicular B-cells (CD19^+^CD21^−^IgM^low^IgD^high^CD23^+^CD93^−^) (23, 24). Both subpopulations were significantly reduced in Sema4C^−/−^ mice (Figures 2C,D). Since the abnormalities in B-cells noted in Sema4C^−/−^ mice appeared to be secondary maturation sites such as peripheral lymphoid tissues rather than in primary maturation sites like bone marrow, we investigated if this impaired maturation was associated with abnormalities in lymphoid morphology. As shown in Figures 2E,F, spleens from Sema4C^−/−^ mice displayed markedly altered structures compared to WT. Sema4C^−/−^ spleens had fewer discreet follicles and there was poor demarcation between T- and B-cell margins. Follicle size in Sema4C^−/−^ mice was significantly larger and follicular appearance was markedly disorganized (Figures 2E,F).

**Table 3:** **Analysis of cell populations in WT and Sema4C^−/^^−^ naive mice in spleens and cervical lymph nodes**.

	Spleen (×10^6^)		Lymph node (×10^4^)	
	WT	Sema4C^−/−^	WT	Sema4C^−/−^
Macrophages	0.66 ± 0.01	0.86 ± 0.23	2.20 ± 0.30	1.70 ± 0.98
Monocytes	2.89 ± 0.31	3.54 ± 0.80	3.08 ± 0.38	3.82 ± 0.25
Dendritic cells	0.21 ± 0.03	0.18 ± 0.05	0.47 ± 0.25	0.15 ± 0.11
Neutrophils	0.02 ± 0.01	0.02 ± 0.01	–	–
Eosinophils	0.07 ± 0.01	0.10 ± 0.02	–	–
CD4^+^ Foxp3^−^ T cells	15.37 ± 1.37	23.14 ± 13.73	4.37 ± 1.46	4.27 ± 1.92
CD4^+^ Foxp3^+^ T cells	1.55 ± 0.07	1.94 ± 0.08	0.43 ± 0.13	0.44 ± 0.14
CD8 T cell	10.32 ± 0.62	11.82 ± 0.46	3.24 ± 0.94	2.76 ± 0.88

*Absolute numbers are presented as indicated*.

*Definition of cell phenoytpes: macrophage: CD3^−^CD19^−^CD11b^+^F4/80^+^; monocytes: CD3^−^CD19^−^Ly6C^+^Ly6G^−^CD11b^+^F4/80^−^; dendritic cells: CD3^−^CD19^−^CD11c^+^MHCII^+^; neutrophils: CD3^−^CD19^−^Ly6C^+^Ly6G^+^CD11b^+^F/480^−^; eosinophils: CD3^−^CD19^−^CD11b^+^SiglecF^+^; CD4^+^Foxp3^+^ T cells: CD3^+^CD4^+^CD19^−^CD11b^−^Foxp3^+^; CD4^+^Foxp3^−^ T cells: CD3^+^CD4^+^CD19^−^CD11b^−^Foxp3^−^; CD8^+^ T cells: CD3^+^CD4^−^CD19^−^CD11b^−^CD8^+^*.

### Sema4C^−/−^ B-Cells Have Impaired Immunoglobulin Production

**Figure 3:**
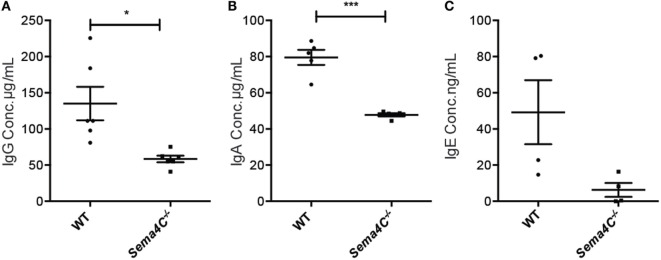
**Sema4C^−/−^ mice demonstrate impaired *in vitro* immunoglobulin production**. Splenic B-cells were isolated from WT and Sema4C^−/−^ mice, then stimulated with anti-CD40 antibodies in the presence of IL-4 and IL-21, at the density of 10^6^ cells/mL. IgG **(A)** and IgA **(B)** concentrations in the supernatant were measured after 4 days of culture by ELISA. IgE **(C)** concentrations were similarly measured by ELISA after 7 days of culture. Means ± SEM, three independent experiments. Each dot represents one individual mouse. *p < 0.05, ***p < 0.001.

Serum IgG levels were comparable between naive Sema4C^−/−^ and WT mice, while serum IgE was not detectable in both naive Sema4C^−^*^/^*^−^ and WT mice (data not shown). We then investigated if the absence of Sema4C would affect immunoglobulin production in cultured B-cells upon Th2 stimulation. B-cells isolated from spleens of Sema4C^−^*^/^*^−^ or littermate WT mice were stimulated with anti-CD40 antibodies, in the presence of IL-4 and IL-21. IgG and IgA concentrations in supernatant were analyzed after 48 and 96 h of stimulation, and IgE was assessed after 7 days of stimulation. As shown in Figures 3A–C, Sema4C^−^*^/^*^−^ B-cells had significantly decreased IgG and IgA production in response to stimulation, as well as lower levels of IgE compared to WT B-cells; however, IgM levels were comparable. Sema4C^−/−^ B cell secreted significantly lower IgG at both 48 and 96 h post activation (Figure S3 in Supplementary Material), although the total number of class switched (CD19^+^sIgM^−^sIgD^−^) B-cells in culture appeared not to differ significantly.

### Sema4C Associates with F-Actin during Murine B-Cell Maturation

**Figure 4:**
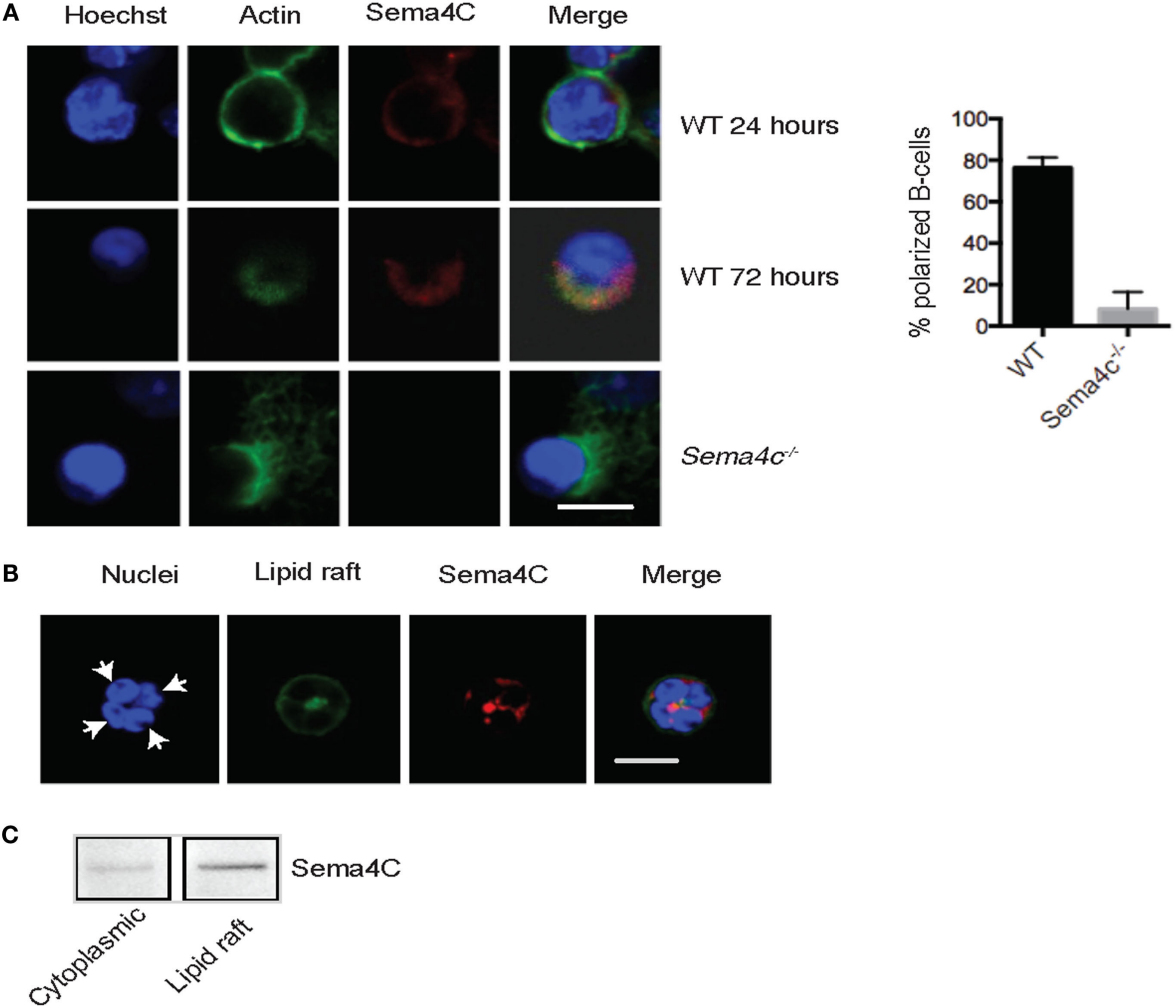
**Murine Sema4C associates with F-actin during B cell maturation**. **(A)** Isolated WT and Sema4C^−/−^ murine splenic B-cells were stimulated with anti-CD40 antibodies, IL-4, and IL-21 in slide chambers and Sema4C (red), F-actin (green), and nuclei (blue) were stained and analyzed by confocal microscopy. Upper panel: 24 h, depicting perimembranous staining of Sema4C and F-actin. Middle panel: WT B-cells, following 72 h of culture, Sema4C and F-actin colocalize toward one pole of the cell. Lower panel: Sema4C^−/−^ following 72 h of culture. No evidence of Sema4C staining and F-actin does not form a normal polar structure. Scale bar: 10 μm. Histogram: percentage of B-cells expressing a synapse-like structure at one pole after 72 h of expression, comparing WT and Sema4C^−/−^ mice. *n* = 3 experiments, total cells counted 3–4 fields with 25 cells in total. **(B)**. Sema4C localizes within lipid rafts. WT B-cells were stimulated with anti-CD40 antibody, in the presence of IL-4 and IL-21. After 72 h, B-cells were double stained with anti-Sema4C antibody (red) and Cholera Toxin B-Alexa Fluor 488 (green). Representative images showing four cells in a cluster with synapse toward the center of the cluster. White arrows indicate one cell, with both the lipid raft fraction and Sema4C located at a pole in the center of the four indicated B-cells. Scale bar: 50 μm. **(C)** B-cells stimulated as in **(B)** were lysed and gradient centrifuged as described in Section “Materials and Methods.” Sema4C was primarily detected in the lipid raft layer (right). Each image represents three independent experiments.

In order to predict potential roles for Sema4C in B-cell differentiation, we compared anti-CD40/IL-4-treated tonsillar B-cells versus unstimulated cells using the **D**atabase for **A**nnotation, **V**isualization and **I**ntegrated **D**iscovery (DAVID) which provided functional annotation clustering of differentially expressed genes (version 6.7, available at https://david.ncifcrf.gov/) (25, 26). Sema4C was placed in three enrichment clusters (Table S2 in Supplementary Material): “membrane,” “intrinsic to membrane,” and “immunoglobulin domain.” Because we observed that follicular development in Sema4C^−/−^ mice was highly abnormal, and DAVID analysis predicted that Sema4C should be an integral component of B-cell membrane dynamics, we therefore evaluated the pattern of membrane expression of Sema4C. Of note, to date no reliable Sema4C antibodies for flow cytometry have been produced. However, suitable antibodies exist for immunohistochemistry and Western blotting. Sema4C was detected on WT B-cells at 24 h following activation with anti-CD40 + IL-4 + IL-21, but not on Sema4C^−/−^ B-cells (Figure 4A, top and bottom panels). Sema4C was distributed uniformly along the membrane of B-cells at 24 h. However, by 72 h, Sema4C expression was strongly localized to a pole or synapse-like structure (Figure 4A, middle panel). Immune synapses are areas which include clusters of signaling complexes and adhesion molecules, critical for organization, and differentiation of B-cells (27). As most immune synapses contain F-actin as an anchor to the coalesced molecules, we costained for F-actin at 24 and 72 h. As seen in Figure 4, Sema4C and F-actin at first colocalize uniformly along the membrane of stimulated B-cells at 24 h, then, following cell polarization, colocalize in a synapse-like structure at 72 h. Importantly, not only was Sema4C protein not present in B-cell synapses following activation in Sema4C^−^*^/^*^−^ mice, but B-cells exhibiting polarization were significantly less frequent (Figure 4). In those cells that did polarize and express a synapse-like structure, staining of F-actin appeared more diffuse and less organized (Figure 4A, lower panel). Taken together with the disrupted architecture found in Sema4C^−/−^ spleens, these data imply that Sema4C may be important in B-cell polarization and the formation of B-cell synapses during follicular development.

Since lipid rafts frequently contain the proteins and surface molecules that migrate to synapses (19–21), we evaluated if Sema4C and lipid rafts colocalized in B-cells using immunofluorescent staining. Lipid rafts were identified by Cholera Toxin B that binds the lipid raft constituent ganglioside GM1. Figure 4B demonstrates a cluster of four B-cells with the lipid raft fraction coalesced toward a pole between the cells. Additionally, Cholera Toxin B staining and Sema4C colocalized on the activated B-cells. This association was confirmed by isolating lipid raft proteins using gradient centrifugation and probing with anti-Sema4C antibodies (Figure 4C).

### Sema4C Colocalizes with F-Actin and Other Key Synaptic Molecules Following Human B-Cell Activation

**Figure 5:**
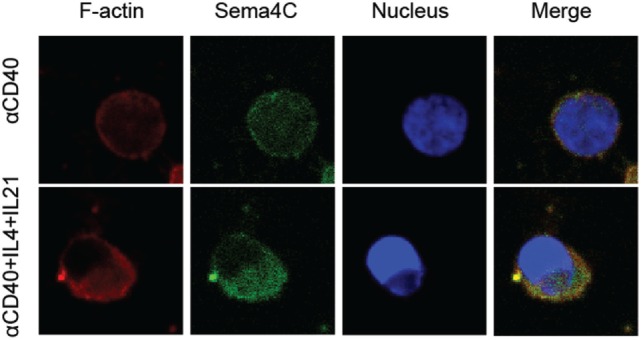
**Human Sema4C associates with F-actin on activated B-cells**. Human B-cells were isolated from tonsils and stimulated with anti-CD40 antibodies ± IL-4 and IL-21 for 5 days in slide chambers. Cells were stained with anti-F-actin antibodies (red), anti-human Sema4C antibodies (green), and a nuclear stain (Hoechst 33342, blue). Upper panel: CD40 antibodies; lower panel: CD40 antibodies + IL-4 + IL-21. Sema4C expression and organization into a synapse-like structure is optimally achieved with anti-CD40 + IL-4 + IL-21 but not with anti-CD40 alone. Representative images of three independent experiments.

Having established the association of Sema4C and F-actin in murine B-cells, we studied whether expression of Sema4C and colocalization with polarized F-actin was also a feature of human B-cell activation. Tonsillar B-lymphocytes were cultured in slide chambers for up to 5 days and Sema4C was visualized *in situ* (5). Similar to murine cells, after 24 h of activation with anti-CD40, IL-4, and IL-21, Sema4C was present in a perimembranous staining pattern. This was also observed with anti-CD40 + IL-4 but not with anti-CD40 alone or anti-CD40 + IFN-γ (data not shown). Upon stimulation for 120 h, B-cells exposed to anti-CD40 + IL-4 + IL-21 exhibited expression of Sema4C localized to one pole of the B-cells (Figure 5, bottom panel). Additionally, as in murine B-cells, Sema4C colocalized with F-actin accumulation. In the absence of Th2 cytokines, there was little expression of Sema4C, polarization or synapse formation (Figure 5, top panel).

**Figure 6:**
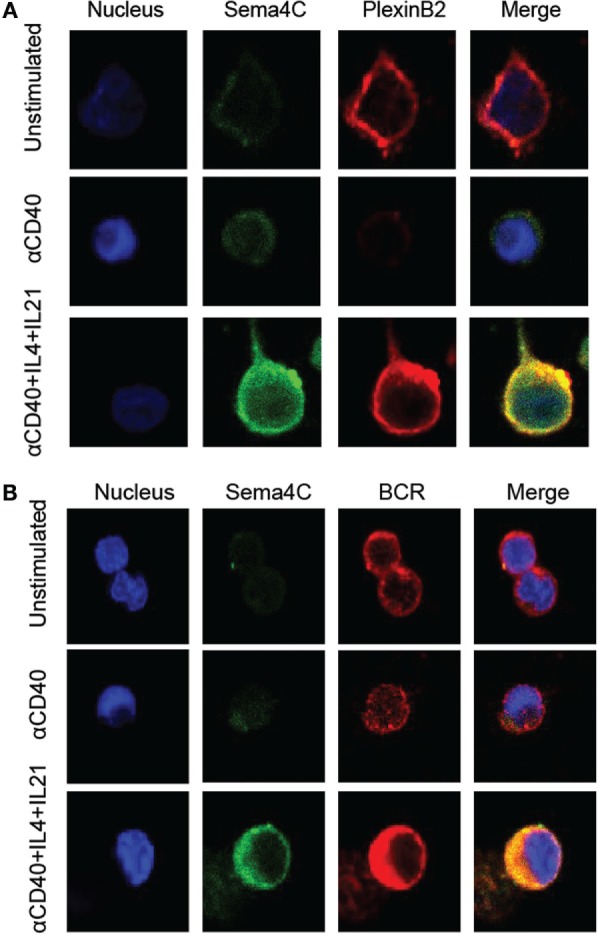
**Human Sema4C colocalizes with Plexin-B2 and BCR**. Human B-cells isolated from tonsil were stimulated with anti-CD40 in the presence or absence of IL-4 and IL-21 for 5 days in slide chamber. **(A)** Representative images of cells stained with anti-Sema4C antibodies (green), anti-Plexin-B2 antibodies (red), and a nuclear stain (Hoechst 33342, blue). Upper panel: unstimulated tonsillar B-cells showing constitutive Plexin-B2 staining but minimal Sema4C; middle panel: anti-CD40 antibodies for 5 days indicating minimal Sema4C expression but no Plexin-B2; and lower panel: anti-CD40 antibodies + IL-4 + IL-21 indicating optimal Sema4C expression and organization into a synapse-like structure colocalizing with Plexin-B2. **(B)** Representative image of cells stained with anti-Sema4C antibodies (green), anti-BCR antibodies (red), and a nuclear stain (Hoechst 33342, blue). Upper panel: unstimulated tonsillar B-cells showing constitutive BCR staining but minimal Sema4C expression; middle panel: anti-CD40 antibodies for 5 days indicating minimal Sema4C expression; and lower panel: anti-CD40 antibodies + IL-4 + IL-21 indicating optimal Sema4C expression and organization into a synapse-like structure colocalizing with BCR.

The primary binding partner for Sema4C, Plexin-B2, is constitutively expressed on a high percentage of resting B-cells in a diffuse membranous pattern (Figure 6A). Following 72–120 h of stimulation, Plexin-B2 and Sema4C were also colocalized within the B-cell clones similar to F-actin (Figure 6A, bottom panel). The approximation of Sema4C and Plexin-B2 at a single pole of activated B-cells was rarely seen in unstimulated B-cells or in the absence of Th2 cytokines (Figure 6A, top and middle panels). The synaptic structure that we identified also included the B-cell receptor (BCR) which is expressed broadly on the membrane in resting cells but is found in the synapse-like pole colocalized with Sema4C following 3–5 days of activation (Figure 6B, bottom panel). In contrast, CD20 was not found to colocalize into the pole but remained diffusely distributed in the membrane (data not shown). In summary, Sema4C is an integral member of the molecules that coalesce to form a synapse-like structure on the surface of B-cells following activation and polarization under Th2 conditions in both murine and human B-cells.

### Sema4C Is Preferentially Induced on CD27^+^ Human Memory B-Cells

We next examined the expression of Sema4C protein within specific populations of activated B-cells. Following anti-CD40 + IL-4, Sema4C expression was detected on approximately 10–15% of cultured B-cells after 72 h. The combination of anti-CD40 + IL-4 + IL-21 induced purified B-cells to express Sema4C at 72 h (Figure S2 in Supplementary Material). The combination of anti-CD40 + IL-4 + IL-21 mediated activation appeared to be specific for induction of Sema4C, as neither BCR stimulation, with or without added Th2 cytokines, nor toll-like 9 receptor stimulation with CpG sequences, increased Sema4C expression (data not shown).

**Figure 7:**
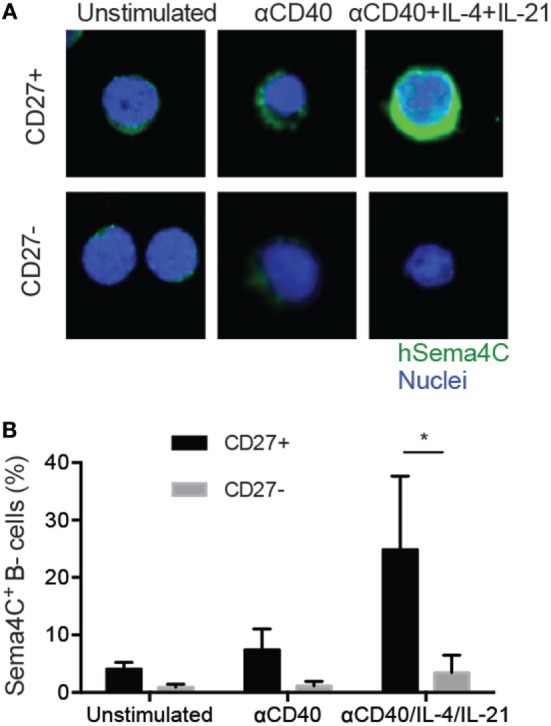
**Sema4C is preferentially induced on memory B-cells**. Isolated human tonsillar B-cells were purified into CD27^+^ and CD27^−^ populations by magnetic bead separation. **(A)** Representative immunofluorescence staining image showing Sema4C protein level in CD27^+^ (upper panel) or CD27^−^ (lower panel) B-cells stimulated with CD40L ± IL-4 and IL-21 for 5 days. Green: Sema4C, blue: nucleus. **(B)**. Histogram summarizing the percentage of Sema4C expressing B-cells among total cells stimulated under indicated conditions. Cells were counted by microscopy by two independent evaluators and means compared. Inter-rater reliability showed a variance of <10%. *p < 0.05, *n* = 8 experiments.

The induction of maximal Sema4C expression by the combination of anti-CD40 + IL-4 + IL-21, which mimics signals from T-follicular helper cells, suggested that Sema4C expression was likely to be on mature, memory B-cells. To determine if this was indeed the case, we fractionated human B-cells into memory and naïve populations, using positive selection for CD27, a marker of human memory B-cells. CD27^+^ and CD27^−^ B-cells were stimulated with anti-CD40 in the presence or absence of IL-4 + IL-21 (28). Direct visualization of Sema4C protein expression by immunohistochemistry indicated that membranous expression of Sema4C was observed in stimulated CD27^+^ B-cells, with significantly fewer CD27^−^ B-cells expressing Sema4C (Figure 7A). As shown in Figure 7B, direct microscopic enumeration of Sema4C+ cells greatly favored CD27^+^ cells over the CD27^−^ cell population. This suggests that Sema4C induction is a feature of Th2-mediated memory B-cell activation.

### Sema4C Induction Is Decreased in Patients with Impaired Immunoglobulin Production

**Figure 8:**
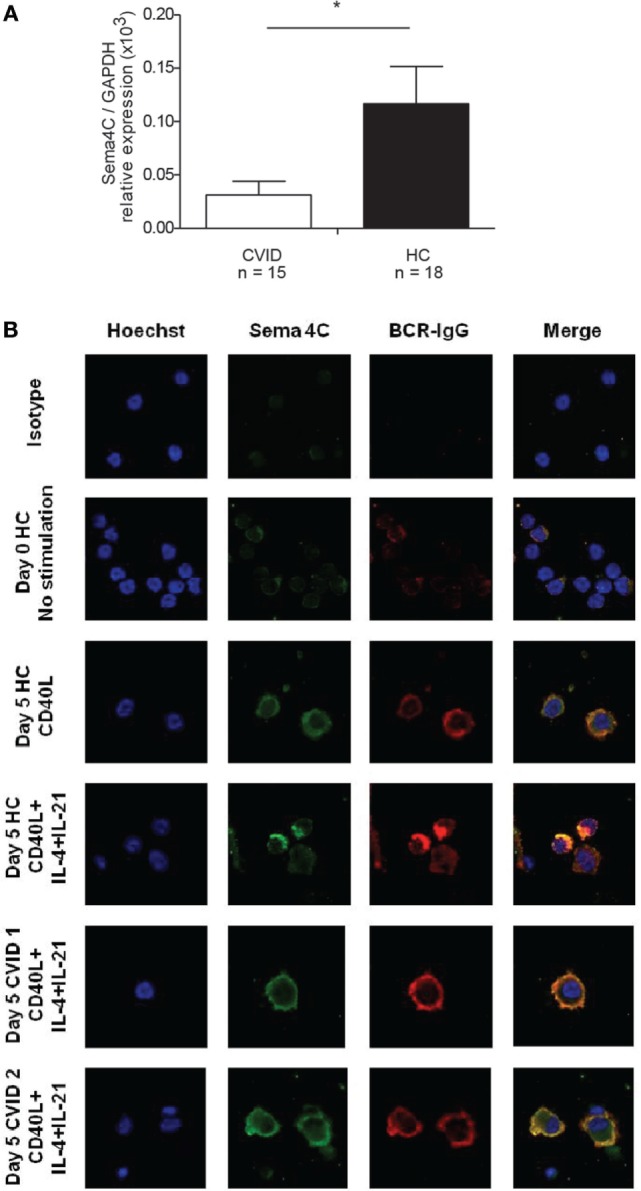
**Patients with common variable immunodeficiency (CVID) have lower Sema4C mRNA and protein expression and impaired synapse-like pole formation**. **(A)** Peripheral blood mononuclear cells (PBMCs) were purified and mRNA extracted. Sema4C mRNA expression was evaluated by semi-quantitative RT-PCR. CVID subjects showed significantly lower basal Sema4C mRNA expression (compared to the GAPDH reference gene) than healthy controls without immune deficiency (HC) (*p = 0.04, Mann–Whitney test). **(B)** PBMC from healthy controls and subjects with CVID were stimulated for 5 days *in vitro* with anti-CD40 antibodies + IL-4 + IL-21. Representative confocal microscopy: HC demonstrated increased expression of Sema4C as well as synapse-like structure formation colocalizing Sema4C with BCR. The two lower panels demonstrate two independently chosen CVID subjects showing membranous Sema4C protein expression and no synapse-like structure formation following 5 days of stimulation with anti-CD40 + IL-4 + IL-21.

To determine if there was an association between Sema4C and impaired B-cell development, we evaluated Sema4C expression in individuals with CVID from a well characterized cohort participating in the C-PRIMES database study for primary immune deficiency (13). Baseline Sema4C mRNA expression was significantly lower in PBMCs from CVID subjects compared to healthy controls (Figure 8A). PBMCs from CVID subjects and controls were then stimulated *in vitro* using anti-CD40 + IL-4 + IL-21, to promote B-cell development and IgG production as well as Sema4C protein expression. CVID subjects had impaired IgG production in culture (mean 1.12 mg/L ± 1.94 SD mg/L) compared to controls (mean 6.31 mg/L ± 5.62 SD mg/L, *p* < 0.001). As observed with tonsillar B-cells, PBMCs from healthy controls demonstrated Sema4C protein expression, polarization, and formation of the synapse-like structure in association with the BCR following 5 days of stimulation with anti-CD40 + IL-4 + IL-21 (Figure 8B). CVID subjects showed some membranous expression of Sema4C protein; however, there was little colocalization with BCR-IgG, and polarization and synapse-like structures were absent, similar to that observed in Sema4C^−/−^ mice. These data suggest that impaired polarization and formation of Sema4C-containing synapse-like structures may be seen of a subset of CVID patients, which may play a role in their diminished Ig production.

## Discussion

Optimal B-lymphocyte development and production of antibodies require coordination, contact and communication between B-cells within clones, and B and T cells within follicles (29). Although molecules such as CD40 and CD40L, integrins, and other costimulatory molecules have been extensively studied, there are still gaps in the understanding of the molecules which mediate exchanges of information by B-cells (30). Our laboratory studies regulatory events in B-cells related to antibody production, immune defects, and severe allergic inflammation (5, 22, 31–34). We have found a novel player which serves a key role in the organization of B-cell follicular structures and optimal antibody production. Semaphorin 4C appears to be required for cellular polarization, and is a component of B-cell synapse formation specifically induced following Th2 cytokine-mediated activation. This appears to be required for normal B-cells differentiation (14).

Using B-cells purified from human tonsils to study events related to induction of IgE-forming B-cells, gene array studies revealed that Sema4C was among the most highly induced genes following 24 h of stimulation (Table 1). As with other class 4 Sema family members, Sema4C includes an extracellular Sema domain, an Ig-like domain, a transmembrane region, and a proline rich intra-cytoplasmic domain (35). Sema4C binds with high affinity to Plexin-B2, a crucial molecule for neural tube closure and brain development in embryogenesis.

Although Sema4C is not the first semaphorin to be found expressed on B-cells, other members of the Sema family such as 4D are constitutively expressed (7) and in our hands were not increased by Th2 activation (Table 2). Sema4C is therefore the first semaphorin specifically induced on B-lymphocytes under Th2 conditions, yet it is among the least explored semaphorins in immune biology. Related family members such as Sema4D and Sema4A have been studied in B-lymphocytes (8, 10), T-lymphocytes (36, 37), and dendritic cells (8, 38). Mice with a selective KO of Sema4D demonstrated poor recall responses to antigens and impaired immunoglobulin production. Interaction of Sema4D-expressing CD5^+^ B-cells or leukemic-B-cells with Plexin-B1-expressing cells led to improved tumor survival. Sema4D is also involved in interaction between T cells and B-cells (39, 40), and in mediating endothelial cell migration (41), while Sema4A is known to activate regulatory T-lymphocytes and cytotoxic T cells (42–44). Interestingly, Sema4A and 4D utilize specific plexins as their primary non-hematopoietic receptors, but appear to interact with different binding partners in the immune system. Sema4A may interact with T-lymphocytes *via* TIM-2, and Sema4D, also known as CD100, is able to diminish BCR signaling and decrease B-cell growth response *via* interaction with CD72 (37). Both T and B-cells can express Sema4D and CD72, potentially leading to mutual regulation signals. To date, no accessory ligands have been determined for Sema4C (2).

B-cell development is predicated upon normal communication between T and B lymphocytes, as well as between B-cells in lymphoid follicles. The points of communication between cells are known as immune synapses (45). The concept of the immune “synapse” has been borrowed from neuroscience, as it describes a defined interface that allows for transmissions of signals between cells. The signals are mediated by cell–cell contact and/or soluble factors, *via* neurotransmitters, cytokines, hormones, or microvesicles. There is much known about the role of T-cell immune synapses in T and B cell interaction B-cell synapses for antigen presentation (29, 46). As suggested by DAVID (Table S2 in Supplementary Material) and STRING analysis (Table S3 in Supplementary Material) we demonstrate that following Tfh-mediated activation, Sema4C is a component in B-cell polarization and B-cell synapses, along with F-actin, the BCR, and Plexin-B2. The paucity of well-defined synapses following B-cell activation in the Sema4C^−/−^ mouse compared to WT may be an important contributor to the lack of formation of normal splenic follicles, as well as decreased numbers of MZ and FOB which were seen in Sema4C^−/−^. In keeping with altered B cell differentiation, production of immunoglobulin *in vitro* is also impaired, suggesting that cell–cell communication within expanding B-cell clones may be malfunctioning.

The optimal conditions for Sema4C upregulation are ligation of CD40 in the presence of IL-4 and IL-21. Ligation of the BCR, of toll-like receptors or other cytokine combinations were unable to upregulate Sema4C (Figure 1; Figure S1 in Supplementary Material and data not shown). This specificity suggests that a principle contact leading to enhanced expression of Sema4C on B-cells is *via* T-follicular helper cells. The primary difference between Tfh and Th2 cell cytokine production is the presence of IL-21 in the Tfh, and the absence of most Th2 cytokines with the exception of IL-4 and IL-21 (47, 48). The interaction between Tfh and B-cells is at the T and B-interface of lymph node follicles. This interaction is tightly regulated by chemokines, is crucial for the productive development of both the Tfh cells and antibody secreting B-cells. Crotty (49) demonstrated that the interaction between B-cells and Tfh is dependent on antigen presentation by B-cells. This may be impaired in mice deficient in Sema4C, as BCR is a component of the immune synapse that forms following CD40 ligation + IL-4 + IL-21. Incomplete focusing of the BCR may lead to decreased antigen presentation, incomplete signaling, and decreased Tfh cell number and function (49). These are important aspects for exploration in our future studies.

The most striking immunological findings in mice deficient in Sema4C are related to B-cell differentiation in the spleen. Specifically, the splenic architecture revealed impaired T-cell/B-cell demarcation, leading to increased follicular size and unusual organization. This disruption in follicular architecture has also been seen in RELB deficient mice (50). This can be due to a role for Sema4C directly *via* interaction with Plexin-B2 expressed on T cells. The potential consequences of impaired B-cell–T-cell communication and follicular abnormalities include diminished subsets of follicular and marginal zone B-cells, which we observed in Sema4C^−/−^ mice. We are using B-cell specific Sema4C^−/−^ mice to study if this molecule is required for Tfh activation and normal humoral responses and response to inhaled allergens.

Interaction between Plexin-B2 and a semaphorin ligand in the process of B-cell maturation was suggested by Yu et al. (51), who determined that Plexin-B2 was expressed by murine and tonsillar germinal center B-cells. Our data suggest that signaling *via* Sema4C and Plexin-B2 in lymphoid tissue thus may be needed for normal Ig production. In our studies, Sema4C was detected almost exclusively in the CD27^+^ fraction on human B-cells, the subset that include predominantly memory B-cells and plasmablasts (52). It is unknown if Sema4C is required for the induction of B-cell memory, but productive GC interactions requiring synapse formation between B and Tfh cells appear to be a prerequisite for normal B-cell memory (53, 54). While we are in the process of evaluating memory and antigen responses in the murine system, we present intriguing preliminary data in individuals with CVID, specifically those with very low memory B-cells and associated poor antibody production, who have impaired upregulation of Sema4C mRNA and impaired synapse formation, analgous to Sema4C^−/−^ mice.

In conclusion, we have determined that a key molecule in the developing nervous system, previously not known to be expressed or functional in immune biology, can be induced in human B-lymphocytes under highly specific conditions. We report expression of Sema4C is induced in human B-lymphocytes, and its expression is found primarily on mature human B-lymphocytes, predominantly on CD27^+^ B-cells. Moreover, the expression of Sema4C appears to be important for normal B-cell polarization following Th2-mediated activation as well as part of a synapse-like structure which includes Plexin-B2. This is the first time Sema4C has been described in human B-cells, and our studies suggest the importance of Sema4C in cell–cell communication regulating B-cell differentiation.

## Author Contributions

DX developed the mouse experimentation, carried out the experiments, and prepared the manuscript. MD and MB performed the human subject data and assisted in preparation of the manuscript. GK performed the gene array analysis and assisted in preparation of the manuscript. SA-T performed the experiment on the cohort of 10 patients. EA provided assistance on the synaptic analysis and assisted in preparation of the manuscript. RF provided the Sema4C mutant mice and valuable technical assistance. WM directed the lipid raft experiments and provided valuable assistance in microscopy synapse analysis. BM conceived of the project and coordinated the research, data analysis, and manuscript preparation. All the authors reviewed and approved the manuscript.

## Conflict of Interest Statement

The authors declare that the research was conducted in the absence of any commercial or financial relationships that could be construed as a potential conflict of interest.
