# Corrigendum: Semaphorin 4C: A Novel Component of B-Cell Polarization in Th2-Driven Immune Responses

**DOI:** 10.3389/fimmu.2017.00213

**Published:** 2017-03-08

**Authors:** Di Xue, Marylin Desjardins, Gabriel N. Kaufman, Marianne Béland, Salem Al-Tamemi, Eisha Ahmed, Shao Tao, Roland H. Friedel, Walid Mourad, Bruce D. Mazer

**Affiliations:** ^1^Translational Research in Respiratory Diseases, The Research Institute of the McGill University Health Center, Montreal, QC, Canada; ^2^McGill University Health Center, Montreal Children’s Hospital, Montreal, QC, Canada; ^3^Sultan Qaboos University Hospital, Muscat, Oman; ^4^Icahn School of Medicine at Mount Sinai, New York, NY, USA; ^5^Department of Medicine, University de Montreal, Montreal, QC, Canada

**Keywords:** semaphorin 4C, B-cells, immune synapse, Th2 responses

There was a mistake in the name of the author Salem Al-Tememi as published. The correct spelling of the name should be Salem Al-Tamemi. The authors apologize for the mistake. This error does not change the scientific conclusions of the article in any way.

In the original article, we neglected to thank Immunodeficiency Canada for providing grant support for part of the study *via* the Chaim Roifman Award Program. The correct Funding section is below.

## Conflict of Interest Statement

The authors declare that the research was conducted in the absence of any commercial or financial relationships that could be construed as a potential conflict of interest.

