# Supplementary material for: Semaphorin 4C: A Novel Component of B-Cell Polarization in Th2-Driven Immune Responses
Source: Front Immunol. 2016 Dec 7;7:558. doi: 10.3389/fimmu.2016.00558 (PMC5141245; doi:10.3389/fimmu.2016.00558)
Supplement: Supplementary file 1 [file Image_1.PDF]

Supplemental Figures

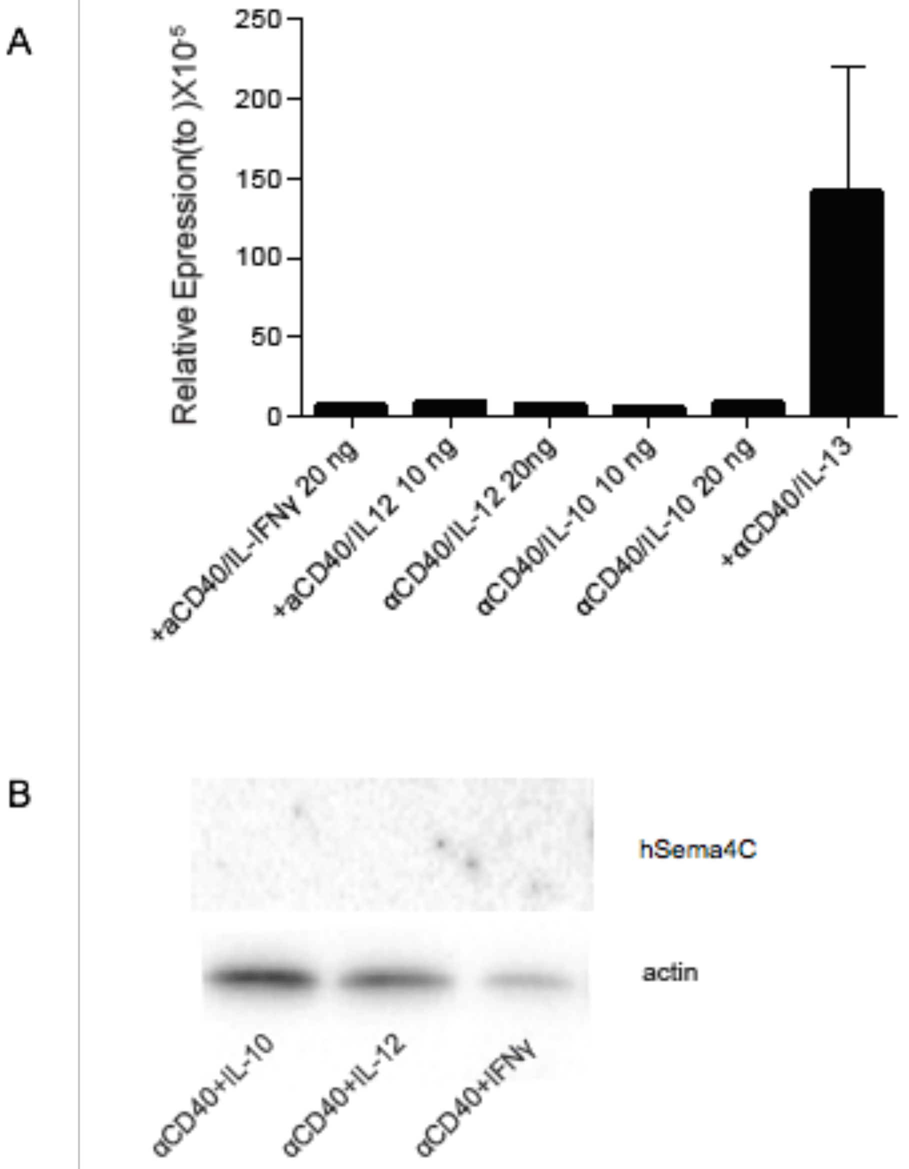

Supplemental Figure1. Sema4C induction on B cells is specific to Th2 cytokines.
Human tonsilar B cells were isolated by RosetteSep Human B cell Enrichment Cocktail, and stimulated with indicated conditions. (**A**) After 24 hours, Sema4C mRNA level was measured by qPCR analysis. Sema4C expression was normalized to RPL27. Means ± SEM, n=10. (**B**) After 5 days, Sema4C protein level in B cells was measured by western blot. Representative picture of at least 3 independent experiments.
