# Supplementary material for: Semaphorin 4C: A Novel Component of B-Cell Polarization in Th2-Driven Immune Responses
Source: Front Immunol. 2016 Dec 7;7:558. doi: 10.3389/fimmu.2016.00558 (PMC5141245; doi:10.3389/fimmu.2016.00558)
Supplement: Supplementary file 2 [file Image_2.PDF]

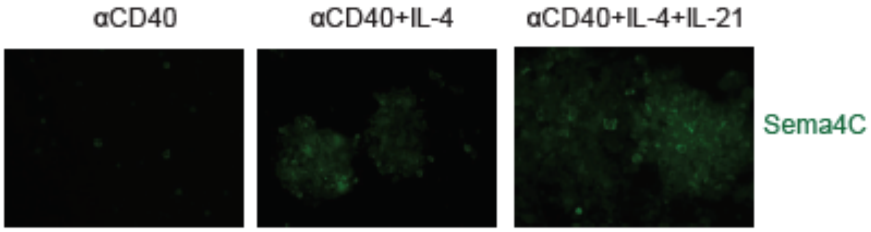

Supplemental Figure2. Sema4C expression in B cells is enhanced by IL-21.
Human tonsilar B cells were isolated by RosetteSep Human B cell Enrichment Cocktail, and stimulated with anti-CD40, anti-CD40+IL-4, or anti-CD40+ IL-4+IL-21 in 8-well slide chambers. After 120 hours, Sema4C expression in B cells was analyzed by immunofluorescent staining. Green: Sema4C. Representative pictures of 3 independent experiments.
