# Supplementary material for: Semaphorin 4C: A Novel Component of B-Cell Polarization in Th2-Driven Immune Responses
Source: Front Immunol. 2016 Dec 7;7:558. doi: 10.3389/fimmu.2016.00558 (PMC5141245; doi:10.3389/fimmu.2016.00558)
Supplement: Supplementary file 3 [file Image_3.PDF]

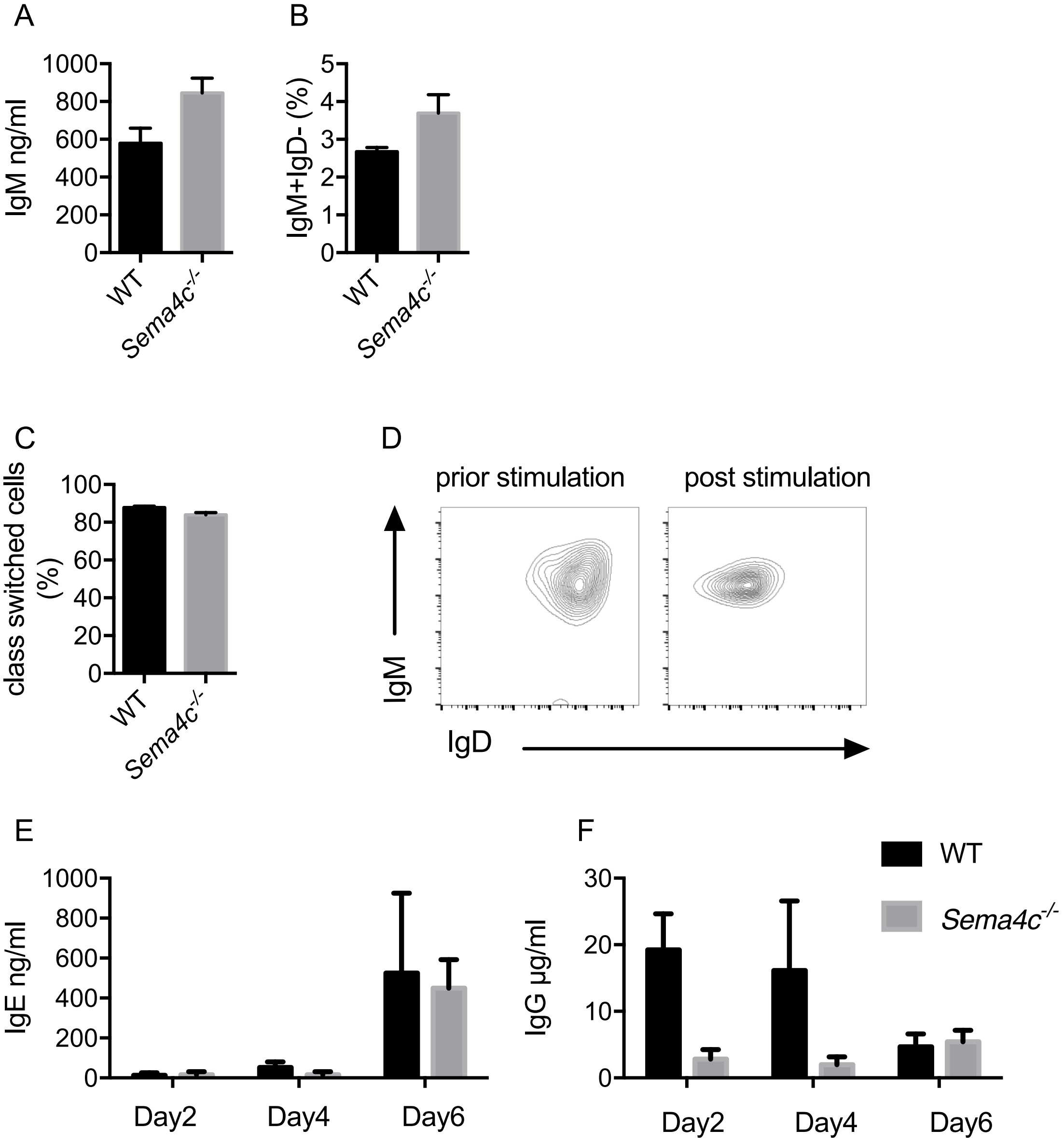

Supplemental figure 3. WT and *Sema4c*$^{-/-}$ B cells were isolated from spleen and stimulated with anti-CD40 in the presence of IL-4 and IL-21. Media was replaced with fresh medium on days 3 and 5 (A) After 3 days, IgM in the supernatant were measured using ELISA. (B) Percentage of IgM single positive cells among total B-cells after 3-days of stimulation. (C) Percentage of IgM^- IgD^- cells among total B cells on day 3, measured by flow cytometry. (D) Representative plots showing IgM and IgD expression before and after stimulation. (E and F) IgE and IgG production from WT and *Sema4c*$^{-/-}$ B cells cultured for indicated times.
