# Supplementary material for: Semaphorin 4C: A Novel Component of B-Cell Polarization in Th2-Driven Immune Responses
Source: Front Immunol. 2016 Dec 7;7:558. doi: 10.3389/fimmu.2016.00558 (PMC5141245; doi:10.3389/fimmu.2016.00558)
Supplement: Supplementary file 4 [file Table_1.PDF]

Supplemental table1: Sequences and annealing temperature of primers used for qPCR.

	Forward	Reverse	Annealing temperature
Human Sema4C	CCATGAGAAGCCTGGTGCTA	CAGTCTGCACAGGAGCGATA	60 °C
Human RPL27	ATCGCCAAGAGATCAAAGATAA	TCTGAAGACATCCTTATTGACG	57 °C
Human Gapdh	CTCTCTGCTCCTCCTGTTCGAC	TGAGCGATGTGGCTCGGCT	62 °C
