# Supplementary material for: Semaphorin 4C: A Novel Component of B-Cell Polarization in Th2-Driven Immune Responses
Source: Front Immunol. 2016 Dec 7;7:558. doi: 10.3389/fimmu.2016.00558 (PMC5141245; doi:10.3389/fimmu.2016.00558)
Supplement: Supplementary file 5 [file Table_2.PDF]

Supplemebtal table2: DAVID functional annotation clustering of differentially expressed genes in anti-CD40/IL4-treated B cells versus unstimulated cells. Statistically significant enrichment terms presented.
Enrichment score: geometric mean (in -log scale) of member's Fisher's Exact test P-values in the annotation cluster
Category: Annotation parent source for ontology terms
Term: Individual enriched ontology terms associated with gene list
Count: number of genes in list that match annotation term
%: Number of genes involved in given term divided by total number of input genes
PValue: from Fisher's Exact test for gene enrichment in annotation terms
Genes: Genes from gene list that are grouped under a specific ontology
List Total: number of genes in gene list mapped to any term in this ontology
Pop Hits (Population hits): number of genes with this GO term on the whole array
Pop Total (Population total): number of genes on the background list mapped to any term in this ontology
Fold Enrichment: ratio of enrichment proportion over background enrichment
Benjamini: Multiple-comparisons corrected P-value for Fisher's Exact test

Annotation Cluster 1	Enrichment Score: 2.456826371375816									
Category	Term	Count	%	PValue	Genes	List Total	Pop Hits	Pop Total	Fold Enrichment	Benjamini
SP_PIR_KEYWORDS	membrane	102	4.700460829	1.24E-04	ADCY1, SCN3A, IL6ST, UTRN, PDE3B, SIDT1, RELL1, VCL, IL17RB, GNG8, S1PR1, CLEC4A, FAS, RNF149, RAPGEF2, AIG1,	229	6256	19235	1.369494019	0.034805415

					TMEM200A, PSD3, CYP26A1, SSPN, METTL7A, CHPT1, CCR6, LPAR5, IGSF3, MGAT5, ADD3, ORAI2, IFITM1, CYSLTR1, FCER2, MMD, TMEM63A, KMO, BCL2L1, EPHB1, AHRR, CNR1, SLC30A4, NDRG1, B4GALT5, ADAM28, GPR155, SCD, TGFBR2, ABCB1, SLAMF1, HOMER2, ABCB4, CD55, SYNE2, PRICKL					

					E1, CD58, TRAF3IP3, SYT17, SLC20A1, IL21R, FCRL2, FCRL1, FCRL5, FCRL4, SPINT2, CXCR4, SNTB1, CHST15, DENND5B, IL13RA1, SLC1A1, RHOF, NT5E, DPEP2, GAPT, ICAM1, BTNL9, CLMN, PRKCB, UGT2B17, CD80, CLIC6, SEMA4C, FAIM3, C16ORF54, GRAMD1C, PMEPA1, LCP1, SLC37A3, HK2, RSAD2, IL7R, SEC62,					

					CD9, GNPTAB, GLIPR1, QSOX1, LY75, CR2, MAL, C10ORF128, FCGR2B, PECAM1, ALOX5, FAM26F					
GOTERM_CC_FAT	GO:0031224~intrinsic to membrane	87	4.00921659	0.001336974	ADCY1, SCN3A, SLC20A1, IL6ST, IL21R, FCRL2, FCRL1, PDE3B, SIDT1, FCRL5, RELL1, FCRL4, IL17RB, S1PR1, SPINT2, CXCR4, CLEC4A, RNF149, FAS, CHST15, IL13RA1, RAPGEF2, DENND5B, NT5E, SLC1A1,	157	5485	12782	1.291343502	0.040411697

					DPEP2, GAPT, ICAM1, BTNL9, NCF2, CLMN, AIG1, TMEM200A, SSPN, CHPT1, UGT2B17, CCR6, CD80, LPAR5, IGSF3, CLIC6, SEMA4C, FAIM3, C16ORF54, GRAMD1C, MGAT5, PMEPA1, ORAI2, CYSLTR1, IFITM1, FCER2, SLC37A3, TMEM63A, MMD, KMO, BCL2L1, IL7R, SEC62, EPHB1, CD9, GNPTAB, GLIPR1,					

					CNR1, SLC30A4, QSOX1, B4GALT5, LY75, ADAM28, GPR155, IL6, CR2, SCD, TGFBR2, MAL, ABCB1, C10ORF128, SLAMF1, ABCB4, CD55, SYNE2, FCGR2B, CD58, PECAM1, TRAF3IP3, SYT17, FAM26F, KCTD12					
Annotation Cluster 2	Enrichment Score: 2.4524974699793276									
Category	Term	Count	%	PValue	Genes	List Total	Pop Hits	Pop Total	Fold Enrichment	Benjamini

SP_PIR_KEYWORDS	Immunoglobulin domain	17	0.783410138	1.63E-04	ICAM1, BTNL9, IL6ST, VPREB3, FCRL2, FCRL1, PALLD, FCRL5, SLAMF1, FCRL4, FCGR2B, CD80, CD58, IGSF3, PECAM1, SEMA4C, FAIM3	229	470	19235	3.038139924	0.022937931
