# Supplementary material for: Semaphorin 4C: A Novel Component of B-Cell Polarization in Th2-Driven Immune Responses
Source: Front Immunol. 2016 Dec 7;7:558. doi: 10.3389/fimmu.2016.00558 (PMC5141245; doi:10.3389/fimmu.2016.00558)
Supplement: Supplementary file 6 [file Table_3.PDF]

Supplemental Table 3

String Database enrichment analysis for Gene Ontology (GO) biological processes and cellular components in the gene network of Sema4C. Top 76 genes identified with FDR ≤0.01.

Legend:
GO pathway ID: Accession number of GO term in the GO database
Pathway Description: Name of GO term animation
Observed Gene Count: Number of genes per GO pathway observed in the gene network
False Discovery Rate (FDR): Multiple Comparisons adjusted p-value for the statistical test of observed gene count in the set compared the genome wide background enrichment.

GO pathway ID	Pathway Description	Observed Gene Count	FDR
GO:0097120	Receptor localization to synapse	4	0.00000355
GO:0007409	Angiogenesis	9	0.0000111
GO:0030182	Neuron differentiation	11	0.0000111
GO:0031175	Neuron projection development	10	0.0000111
GO:0035418	Protein localization to synapse	4	0.0000111
GO:0061564	Axon development	9	0.0000111
GO:0048667	Cell morphogenesis involved in neuron differentiation	9	0.0000119
GO:0048812	Neuron projection morphogenesis	9	0.0000128
GO:0071526	Semaphorin-plexin signaling pathway	4	0.000015
GO:0048666	Neuron development	10	0.0000157
GO:0050772	Positive regulation of axonogenesis	5	0.0000157
GO:0000904	Cell morphogenesis involved in differentiation	9	0.0000397
GO:0040011	Locomotion	11	0.0000397
GO:0030030	Cell projection organization	10	0.0000789
GO:0006928	Movement of cell or subcellular component	11	0.0000871
GO:0048699	Generation of neurons	11	0.0000871
GO:0048858	Cell projection morph	10	0.0000874
GO:0032990	Cell part morphogenesis	9	0.0000938
GO:0010976	Positive regulation of neuron projection development	6	0.0000999
GO:0022008	Neurogenesis	11	0.000114
GO:0043113	Receptor clustering	4	0.000114
GO:0007411	Axon guidance	7	0.000178
GO:0050770	Regulation of axonogenesis	5	0.000268
GO:0000902	Cell morphogenesis	9	0.000364
GO:0031346	Positive regulation of cell projection organization	6	0.000364
GO:0048468	Cell development	11	0.000364
GO:0045666	Positive regulation of neuron differentiation	6	0.000448

GO:0010770	Positive regulation of cell morphogenesis involved in differentiation	5	0.000495
GO:0030154	Cell differentiation	14	0.000659
GO:0016477	Cell migration	8	0.000689
GO:0016310	Phosphorylation	9	0.000877
GO:0010975	Regulation of Neuron projection dev	6	0.000915
GO:0046939	Nucleotide phosphorylation	4	0.000923
GO:0048869	Cellular dev process	14	0.00101
GO:0048870	Cell motility	8	0.00112
GO:0051674	Localization of cell	8	0.00112
GO:0050769	Positive regulation of neurogenesis	6	0.0012
GO:0045197	Establishment or maintenance of epithelial cell apical/basal polarity	3	0.00125
GO:0050803	Regulation of synapse structure or activity	5	0.00175
GO:0007399	Nervous system development	11	0.00179
GO:0006935	Chemotaxis	7	0.00195
GO:0035088	Establishment or maintenance of apical/basal cell polarity	3	0.00195
GO:0042330	Taxis	7	0.00195
GO:0061245	Establishment or maintenance of bipolar cell polarity	3	0.00195
GO:0016043	Cellular component organization	16	0.00197
GO:0009653	Anatomical structure morphogenesis	11	0.00255
GO:0071840	Cellular component organization or biogenesis	16	0.00262
GO:0060429	Epithelium development	8	0.00296
GO:0031344	Regulation of cell projection organization	6	0.00313
GO:0010720	Positive regulation of cell development	6	0.00351
GO:0048167	Regulation of synaptic plasticity	4	0.0041
GO:0045664	Regulation of neuron differentiation	6	0.00427
GO:0007423	Sensory organ development	6	0.0057
GO:0010769	Regulation of cell morph involved in differentiation	5	0.00593
GO:0045597	Positive regulation of cell differentiation	7	0.00723
GO:0060562	Epithelial tube morphogenesis	5	0.00836
GO:0018108	Peptidyl-tyrosine phosphorylation	4	0.00878
GO:0018212	Peptidyl-tyrosine modification	4	0.00909
GO:0021915	Neural tube development	4	0.00949
GO:0048598	Embryonic morphogenesis	6	0.00949
GO:0035295	Tube development	6	0.011
GO:0007166	Cell surface receptor signaling pathway	10	0.016
